# Effect of multiple drug resistance on total medical costs among patients with intra-abdominal infections in China

**DOI:** 10.1371/journal.pone.0193977

**Published:** 2018-03-28

**Authors:** Xuemei Zhen, Yuanyuan Li, Yixi Chen, Peng Dong, Stephanie Liu, Hengjin Dong

**Affiliations:** 1 Center for Health Policy Studies, School of Public Health, Zhejiang University School of Medicine, Hangzhou City, Zhejiang Province, China; 2 Pfizer Investment Co. Ltd., Beijing, China; National Yang-Ming University, TAIWAN

## Abstract

**Background:**

Multiple drug resistant (MDR) intra-abdominal infections (IAIs) are associated with notable direct and societal costs. As previous studies have not considered the impact of MDR on the total medical costs (TMCs) of IAIs, the present one examines this, as well as further estimates the additional costs at a national level.

**Methods:**

This is a retrospective study. Firstly, we randomly selected a sample of 40% of all inpatients discharged between 2014 and 2015 from a teaching hospital, due to limits in budget and the large number of patients. Then, we manually selected 254 patients with IAIs according to the International Classification of Disease, 10^th^ revision, using electronic medical records. Eventually, 101 patients with IAIs (64 MDR patients and 37 non-MDR patients) were included after excluding cases without laboratory test results, any pathogens detected, or antimicrobial resistant pathogens. Univariate analysis and a generalized linear model were applied to assess the parameters associated with TMCs.

**Results:**

Compared to non-MDR patients, those with MDR pathogens were significantly associated with higher TMCs, higher antimicrobial costs, higher antimicrobial usage, larger number of pathogens, and longer length of stay and were more likely to have insurance and combination antimicrobial therapy. In addition, the average TMC among patients with MDR pathogens was ¥ 131801, which is ¥ 90201 higher than those without MDR pathogens. If our results are applied to the whole country, the sum of all attributable TMCs was ¥ 37 billion. The societal costs, furthermore, were ¥111 billion in 2015.

**Conclusion:**

Our results provide information that should lead to increased efforts to reduce inappropriate antimicrobial therapy, in order to decrease the emergence of MDR pathogens and to reduce their economic burden.

## Introduction

Intra-abdominal infections (IAIs) represent a vast variety of diseases, including infections of single organs (e.g. cholecystitis, appendicitis, diverticulitis, cholangitis, pancreatitis, and salpingitis), peritonitis, and intra-abdominal abscesses [1,2]. Inpatients with IAIs often possess infections that are dangerous, abnormal, and accompanied by significant mortality; complicated IAIs are associated with 10.5% of mortality worldwide [3].

Patients with both complicated and uncomplicated IAIs may be commonly treated with antimicrobial therapy [4]. Inappropriate antimicrobial therapy may give rise to poor patient outcomes, such as higher mortality, longer hospitalization, and increased economic burden. Furthermore, there is an increased risk that future antimicrobial therapy will be less effective for both previous and new inpatients, because of antimicrobial resistance (AMR) [1].

AMR occurs when medications used to cure infections become ineffective [5]. AMR includes multiple drug resistance (MDR), resistance to three or more types of antimicrobial agents and non-MDR, and resistance to less than three types of antimicrobial agents [6]. Inappropriate antimicrobial usage is widespread and contributes to the rapid spread of AMR pathogens, particularly in developing countries [7]. It is widely accepted that there is a direct association between antimicrobial usage and the development of AMR, which poses significant risks to human health. In 2014, the World Health Organization (WHO) announced the coming of a post-antibiotic era, in which common infections and minor injuries that had been treatable for decades can once again spread and kill [8]. It was estimated that 10 million deaths each year would occur worldwide, with one million deaths occurring in China in 2050. Additionally, the global costs were estimated to be $100 trillion USD, with costs to China accounting for $20 trillion USD [9].

Recently, AMR is becoming more complicated and MDR pathogens are increasingly serious. There is a dramatically increasing trend in the incidence of IAIs caused by MDR pathogens, which creates a major challenge in the treatment of IAIs [10]. In the past few decades, a growing number of MDR pathogens have been detected from IAIs, including methicillin-resistant *Staphylococcus aureus* (MRSA), carbapenem-resistant *Pseudomonas aeruginosa* (CRPA), extended-spectrum *β-lactamase* (ESBL)-producing *Escherichia coli* or *Klebsiella pneumoniae*, carbapenem-resistant *Enterobacteriaceae* (CRE) and carbapenem-resistant *Acinetobacter baumannii* (CRAB) [11]. In addition, MDR IAIs further increase medical costs, stemming from the prolonged length of stay (LOS) in hospitals and poor patient outcomes because of antimicrobial treatment failures [12].

Many studies have investigated the cost analysis of antimicrobial therapy for treatment of IAIs [13,14,15,16]. Studies on clinical characteristics and pathogen distribution of IAIs have also been conducted [17,18]. However, very few studies have examined the association between MDR and total medical costs (TMCs) among patients with IAIs [19]. Compared to previous studies, the present study takes both AMR and TMCs into consideration, focusing on the impact of MDR on TMCs. This study, conducted at a teaching hospital located in the Zhejiang province of China, aims to compare the TMCs among patients with IAIs between MDR and non-MDR groups, and to estimate the additional costs at a national level in China.

## Methods

### Study site

This study was conducted in a tertiary teaching and general hospital in Hangzhou, Zhejiang Province, China, which has a total of 3200 beds and approximately 121,900 discharged patients each year. We specifically chose this hospital as our study site because it is in the provincial capital city, which is the political and cultural center of Zhejiang province. In addition, a high quality hospital information system (HIS) is existent, which makes the collection of complete and reliable data possible.

### Study design, sample, and sampling

This is a retrospective study. Patients with IAIs and AMR pathogens were included in our analysis, and were divided into MDR and non-MDR groups based on antimicrobial susceptibility testing.

**Fig 1:**
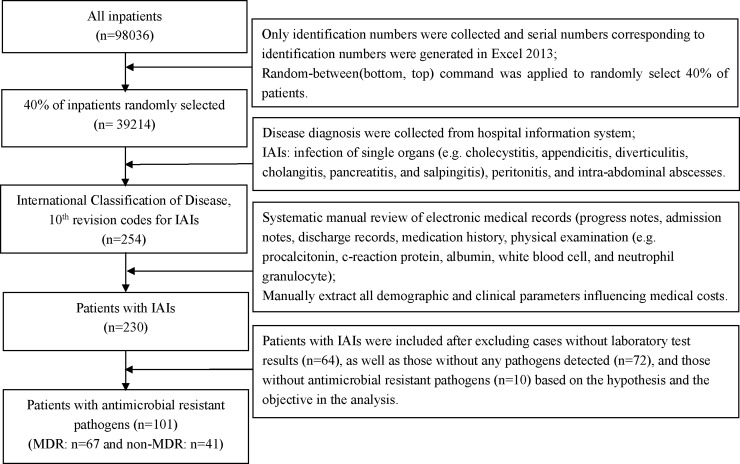
Sample collection procedure.

Firstly, we randomly selected a sample of 40% of all inpatients discharged between 2014 and 2015 in the hospital, due to limiting budget and the large number of patients. Then, two researchers independently manually collected disease diagnosis. Subsequently, we screened in 254 patients according to the International Classification of Disease, 10^th^ revision (ICD-10) codes for IAIs and 230 patients were truly patients with IAIs by systematic manual review of electronic medical records. Moreover, we identified antimicrobial resistant pathogens based on antimicrobial susceptibility testing. Eventually, 101 patients with IAIs were included (64 MDR patients and 37 non-MDR patients) after excluding those without laboratory test results, as well as those without any pathogens detected and those without antimicrobial resistant pathogens based on the hypothesis and the objective in the analysis (Fig 1).

In the sampling, we used the following procedures. Firstly, the identification numbers of all discharged patients were collected from the HIS and serial numbers corresponding to the identification numbers were generated in Excel 2013. Then, random-between (bottom, top) command was applied to randomly select the patients. If one patient has multiple visits, we regarded this patient as multiple unique patients.

### Data collection

Demographic and clinical parameters influencing medical costs were collected from electronic medical records. Any disagreements were resolved with consultation from an experienced physician.

The demographic parameters included identification number, age, sex, medical insurance, and health outcome. Clinical parameters included discharge department, disease diagnosis, susceptibility testing results, antimicrobial usage, surgery, LOS, and ICU admission. Based on the existing parameters, we determined more parameters that may influence medical costs, as follows: combination antimicrobial therapy, carbapenem usage, number of administered antimicrobial agents, and number of pathogens.

The TMC for each patient’s entire admission was calculated. TMC includes ward stays, ICU stays, laboratory tests, medical tests, microbiological cultures, surgical procedures, antimicrobial therapy and other drugs during the period of hospital stay [20]. Since the study was conducted over a span of 2 years, we adjusted TMCs to the year 2015’s currency using the Consumer Price Index for China [20]. The study was approved by the institutional review board of Zhejiang University School of Public Health, and owing to the retrospective nature of the study, informed consent was not deemed necessary. All patient data were anonymized prior to analysis.

### Data analysis

Statistical analyses were performed with the SPSS 17.0 for Windows. The Chi-square test or Fisher exact test was used to analyze categorical parameters. The Student *t-*test was conducted for analyzing age, while a Mann-Whitney *U* test was used for analyzing other parameters of non-normal distribution. Then, the effect of each parameter on the TMCs was evaluated by univariate analysis and a Kruskal-Wallis nonparametric test was employed to assess categorical parameters on TMCs, because it was confirmed to be non-normally distributed. In order to control the effects of parameters on TMCs, a generalized linear model was performed, which included the following parameters: outcome, insurance, age, sex, surgery, combination antimicrobial therapy, carbapenem usage, LOS, ICU admission, number of administered antimicrobial agents, and number of pathogens. Finally, all tests were two-tailed and *P* values less than 0.05 were considered as statistically significant. To correct for the non-normal distribution of the TMCs, a logarithmic transformation was specified.

The parameters in the generalized linear model were selected due to our hypothesis and previous research [17,19,21]. We assumed that TMCs are associated with demographic parameters such as age, sex, insurance, outcome, and clinical parameters such as surgery, combination antimicrobial therapy, carbapenem usage, LOS, ICU admission, number of administered antimicrobial agents, and number of pathogens. Moreover, clinical parameters positively influenced TMCs.

## Results

### Sample characteristics

A total of 101 patients with IAIs caused by AMR pathogens were included, 63.4% of which were MDR patients. The average age of all patients was 57.2 years and most of the patients were men. About half of the patients were covered by insurance, received combination antimicrobial therapy, or admitted to the ICU. Most of them had been previously treated with carbapenems and had undergone surgery. The main outcome (67.3%) upon hospital discharge was an improvement in health (Table 1).

**Table 1:** Characteristics among patients with intra-abdominal infections.

Parameters	Total(n = 101)	MDR(n = 64)	Non-MDR(n = 37)	P-value
Age, mean±SD, years	57.2(20.4)	58.4(20.1)	55.1(20.9)	0.434
Male, (%)	72.3	70.3	75.7	0.562
Insurance, (%)	58.4	50	73	0.024*
Outcome, (%)				0.675
Cure	14.9	12.5	18.9	
Improvement	67.3	68.8	64.9	
Else	17.8	18.8	16.2	
Number of administered antimicrobial agents	3.4(2.1)	4.02(2.3)	2.4(1.3)	<0.000*
Carbapenem usage, (%)	79.2	82.8	73	0.24
Combination antimicrobial therapy, (%)	44.6	56.2	24.3	0.002*
Number of pathogens	2.4(2.1)	3.0(2.4)	1.4(0.6)	<0.000*
Surgery, (%)	81.2	84.4	75.7	0.281
ICU, (%)	61.4	76.6	35.1	<0.000*
LOS, mean±SDb, days	25.4(25.3)	31.0(28.8)	15.59(13.1)	<0.000*

* Significance was determined at the 0.05 level.

SD: standard deviation; LOS: length of stay; ICU: intensive care unit; MDR: multiple drug resistance

Compared to non-MDR patients, a larger number of administered antimicrobial agents, a larger number of pathogens, and a longer LOS were observed in the MDR patients. Additionally, there were significantly more patients in the MDR group who were covered by insurance, received combination antimicrobial therapy, or admitted to the ICU. However, there were no significant differences between the MDR and non-MDR groups with regard to age, sex, carbapenem usage, surgery, and outcome (Table 1).

Pathogens used for analysis were strains collected in the susceptibility tests, with duplicate isolates (the same pathogen from the same patient) being excluded [18,19]. A total of 262 pathogens were detected from 101 patients with IAIs. Out of all pathogens, 35 were non-resistant pathogens, 126 were non-MDR pathogens, and 101 were MDR pathogens. CRAB was the most frequently isolated MDR pathogen, followed by MRSA, ESBL-producing pathogen, CRE, methicillin-resistant coagulase negative *Staphylococci* (MRCNS), methicillin-resistant *Staphylococcus epidermis* (MRSE), and CRPA. For MDR pathogens, the most frequently submitted sample type was sputum, followed by pus and drainage fluid. *Escherichia coli* was the most frequently isolated out of the non-MDR pathogens, followed by *Klebsiella pneumoniae*, *Enterococcus faecium*, *Stenotrophomonas maltophilia*, *Acinetobacter baumannii*, *Enterococcus faecalis*, *Morganella morganii*, and other pathogens (S1 Table). For non-MDR pathogens, drainage fluid was the most common sample type, followed by sputum, ascites, pus, and other types (S2 Table).

### Total medical costs

Patients with MDR pathogens were associated with a mean TMC of ¥ 90201 higher than that of non-MDR patients. Other parameters with a significant association with TMC were age ≤65 years, age >65 years, female, male, insurance, combination antimicrobial therapy, treatment with carbapenems, number of administered antimicrobial agents >3 agents, surgery, LOS >15 days, and admission to the ICU. There were no significant differences in other parameters on TMCs (Table 2).

**Table 2:** Total medical costs.

Parameters	MDR		Non-MDR		P-value
	Mean	SD	Mean	SD	
Total medical costs	131801	135097	41600	31974	<0.000*
Antimicrobial costs	24914	36409	4734	4417	<0.000*
Age					
≤65	112508	12268	35050	32789	0.004*
>65	158249	149229	53693	27608	0.002*
Sex					
Female	135130	129703	40841	38302	0.008*
Male	130396	138721	41844	30466	0.001*
Insurance					
No	118327	121332	35470	30363	0.006*
Yes	145275	148307	43871	32812	0.001*
Outcome					
Cure	74336	58635	23065	16256	0.054
Improvement	129104	140014	46610	35290	0.008*
Else	180001	144025	43184	26655	0.002*
Number of administered antimicrobial agents					
≤3	59219	59671	38982	33706	0.22
>3	195844	150662	52821	21488	<0.000*
Combination antimicrobial therapy					
No	63742	60214	35686	30805	0.101
Yes	184736	153190	59999	29911	0.001*
Carbapenem usage					
No	13024	11575	17695	15149	0.434
Yes	156453	135950	50454	32170	<0.000*
Number of pathogens					
≤1	47105	40948	35249	28144	0.482
>1	176166	146015	53326	36321	<0.000*
Surgery					
No	41120	63109	26922	19128	0.87
Yes	148594	138491	46318	34056	<0.000*
ICU					
No	22225	25364	28688	19805	0.001*
Yes	165345	137355	65438	36962	<0.000*
LOS					
1~	39958	42999	33179	22433	0.906
15~	103428	56477	49172	29606	0.004*
30~	237570	166241	75090	61950	0.017*

All costs are expressed in 2015 constant Chinese Yuan

* Significance was determined at the 0.05 level

SD: standard deviation; LOS: length of stay; MDR: multi drug resistance; ICU: intensive care unit

### Factors influencing total medical costs

In the generalized linear model, in the MDR group, the most significant parameters affecting TMCs for patients with IAIs included admission to the ICU, carbapenem usage, number of pathogens, and LOS. The most influential parameter was admission to the ICU. Moreover, in the non-MDR group, the most significant parameters affecting TMCs for patients with IAIs included carbapenem usage, admission to the ICU, number of administered antimicrobial agents, LOS, and age. Carbapenem usage was the most influential parameter. In the MDR group, we found that TMCs among patients with carbapenem usage, or admission to the ICU were 2.42 times or 2.52 times as high as that among patients without, respectively. TMCs increased by 1.09 times or 1.01 times when the number of pathogens increased by one pathogen or LOS increased by one day, respectively. In the non-MDR group, we found that TMCs among patients with carbapenem usage or admission to the ICU were 1.55 times or 1.50 times of that among patients without, respectively. TMCs increased by 1.17 times, 1.11 times or 1.02 times when the number of administered antimicrobial agents increased by one agent, LOS increased by one day, or the age increased by one year (Table 3).

**Table 3:** Factors influencing total medical costs.

Parameters		MDR					Non-MDR				
		b	SD	95% CI		P-value	b	SD	95% CI		P-value
	(Intercept)	9.20	0.31	8.58	9.81	<0.000*	7.63	0.34	6.95	8.30	<0.000*
Sex	Male	-0.03	0.14	-0.30	0.25	0.859	0.27	0.16	-0.04	0.59	0.090
	Female	0					0				
Insurance	Yes	-0.16	0.13	-0.41	0.09	0.215	0.03	0.17	-0.31	0.37	0.864
	No	0					0				
Outcome	Else	-0.19	0.24	-0.67	0.29	0.431	-0.13	0.26	-0.64	0.38	0.616
	Improvement	-0.36	0.20	-0.75	0.02	0.066	-0.19	0.21	-0.60	0.21	0.351
	Cure	0					0				
Combination antimicrobial therapy	Yes	0.22	0.15	-0.08	0.52	0.145	0.04	0.21	-0.36	0.45	0.836
	No	0					0				
Carbapenem usage	Yes	0.89	0.32	0.26	1.51	0.005*	0.44	0.17	0.10	0.77	0.011*
	No	0					0				
Surgery	Yes	-0.16	0.23	-0.62	0.29	0.485	0.30	0.18	-0.07	0.66	0.109
	No	0					0				
ICU	Yes	0.93	0.28	0.39	1.47	0.001*	0.40	0.16	0.08	0.72	0.013*
	No	0					0				
Age		0.00	0.00	0.00	0.01	0.269	0.02	0.00	0.01	0.03	<0.000*
Number of administered antimicrobial agents		0.04	0.04	-0.03	0.11	0.262	0.15	0.07	0.02	0.29	0.023*
Number of pathogens		0.08	0.03	0.02	0.15	0.008*	0.14	0.12	-0.11	0.38	0.367
LOS		0.01	0.00	0.01	0.02	<0.000*	0.11	0.12	-0.12	0.34	<0.000*

*Significance was determined at the 0.05 level

MDR: multi-drug resistant; SD: standard deviation; CI: confidence interval; ICU: intensive care unit; LOS: length of stay

### Total medical costs in whole country

The mean TMC difference between the MDR and non-MDR groups was ¥ 90201. The rates of MDR IAIs (number of patients with MDR IAIs / number of patients with IAIs) or non-MDR IAIs (number of patients with non-MDR IAIs / number of patients with IAIs) were 27.83% or 16.09%, respectively. Additionally, the rate of IAIs (number of patients with IAIs / number of patients) was 0.587%. In China, there were nearly 1,235,870 cases of IAIs, including 343,943 cases with MDR pathogens and 198,851 cases with non-MDR pathogens [22]. If our results are applied to the whole country, the sum of all attributable TMC was ¥ 37 billion. The societal costs, furthermore, were ¥ 111 billion in 2015, based on WHO suggestion that the societal costs may be more than 3 times the direct costs caused by AMR [23].

## Discussion

Few studies have investigated the impact of MDR on TMCs among patients with IAIs, despite its heavy burden on the Chinese health system. One study focused on the ESBL-producing pathogens but the published data were from 2000 [19]. To our knowledge, our study is the first investigation to provide an overall view of the attributable costs of MDR IAIs in China. Moreover, this is one of few real-world data analyses on Chinese patients, especially in the face of the emergence and spread of MDR pathogens in China.

Our study reveals that MDR is an important driver of TMCs, as results show that MDR inpatients with IAIs are associated with larger number of administered antimicrobial agents, larger number of pathogens, and less likely to have insurance, which are the factors contributing mostly to both TMCs and antimicrobial costs. Several systematic reviews suggest that TMCs attributable to MDR pathogens [24] and due to IAIs [25,26] were considerably high. Moreover, Bijie Hu et al. found that IAIs caused by ESBL-positive producing pathogens were significantly associated with longer LOS, higher TMCs and higher antimicrobial costs [19].

In generalized linear model, it was also noted that admission to the ICU has a strong positive correlation with TMCs. Indeed, the majority of previous studies have shown that ICU patients are more likely to undergo surgery and receive more kinds of antimicrobial agents [27,28]. All the above factors could lead to acquiring resistant pathogens. A reduction in admissions to the ICU may improve inpatient safety, in addition to increasing the quality of care and potentially hospital turnover, and reducing economic burden.

It is known that patients with MDR pathogens are associated with longer LOS compared with patients with non-MDR pathogens, which suggests that differences in costs may be related to LOS. Some studies also indicated that even a one day reduction in hospitalization may result in substantial cost-savings [29,30].

Patients with MDR pathogens were associated with a significant increase in TMCs, because they were treated with carbapenems, admitted to the ICU, stayed longer in hospital, and had more pathogens present. Carbapenems are often considered as drugs of a last resort in strategies for MDR infections, and exposure to carbapenems was associated with carbapenem resistance [31,32]. Moreover, empirical antimicrobial therapy is essential to improving patient outcomes, but it could potentially lead to unnecessary exposure to broad-spectrum antimicrobial agents [33], resulting in MDR and thus increasing TMCs [34]. In addition, the emergence of MDR pathogens can also lead to higher frequencies of failure for empirical antimicrobial therapy, resulting in even higher TMCs [34].

In China, there is a trend of AMR increasing across multiple antimicrobial agents. Between 2005 and 2014, rates of ESBL-producing *Escherichia coli* were between 51.7% and 55.8%. Carbapenem resistance among *Klebdiella pneumonia* and *Acinetobacter baumannii* increased from 2.4% to 13.4%, and from 31% to 66.7%, respectively [35]. In this study, IAIs were mostly caused by *Escherichia coli* and *Klebsiella pneumoniae*, which is consistent with other studies. However, it has been revealed that these pathogens often fail to be treated with various *β-lactam* agents but carbapenems continue to retain activity [36]. Moreover, this study has a higher proportion of patients with CRAB, compared to other pathogens, indicating that the efficacy of carbapenems against ESBL-producing pathogens is decreasing and CRAB is becoming an increasingly serious matter [37].

Several limitations are worth noting. Firstly, due to the nature of retrospective study, we cannot distinguish colonization or infection, which incur significantly different costs. IAIs, however, unlike bloodstream infection or pneumonia, have less chance of colonization. Secondly, future studies should be carried out to directly estimate the societal costs. In this study, we only estimated direct medical costs, and used the relationship between medical costs and societal costs suggested by WHO to estimate the societal costs. This estimate may overestimate the economic burden and may not apply to the Chinese situation nationally. In addition, since this study was conducted only in one tertiary teaching and general hospital in Zhejiang province, generalization of the direct costs to a national level may be overestimated. Thus, multicenter and prospective studies are needed in the future. Lastly, the Acute Physiology and Chronic Health Evaluation II score is a powerful instrument for stratifying risk that is often used for evaluating the severity of patients [2], which was not obtainable in the present HIS.

## Conclusions

This retrospective study shows the heavy burden associated with MDR among patients with IAIs. We conservatively estimate that a large amount of medical costs per hospitalization can be saved, if MDR pathogens can be substantially reduced. The enormous economic burden imposed by MDR IAIs should attract the attention of decision makers in various fields. Moreover, our results provide information that should lead to increased efforts to reduce inappropriate antimicrobial therapy in clinical practice, in order to prevent the emergence and spread of MDR pathogens and to further reduce their economic burden.

## Supporting information

S1 Table Isolation of pathogens. (DOCX)

S2 Table Isolation of sample types. (DOCX)
