# Supplementary material for: Effect of multiple drug resistance on total medical costs among patients with intra-abdominal infections in China
Source: PLoS One. 2018 Mar 28;13(3):e0193977. doi: 10.1371/journal.pone.0193977 (PMC5873998; doi:10.1371/journal.pone.0193977)
Supplement: S1 Table — (DOCX) [file pone.0193977.s001.docx]

| S1 Table. Isolation of pathogens |  |
| --- | --- |
| Pathogens | No. of isolates |
| Non-resistance | 35 |
| *Monilia albicans* | 12 |
| *Escherichia coli* | 9 |
| *Candida tropicalis* | 7 |
| *Candida parapsilosis* | 3 |
| Else | 4 |
| Non-multiple drug resistance | 126 |
| *Escherichia coli* | 21 |
| *Klebsiella pneumoniae* | 17 |
| *Enterococcus faecium* | 12 |
| *Stenotrophomonas maltophilia* | 9 |
| *Acinetobacter baumannii* | 8 |
| *Enterococcus faecalis* | 6 |
| *Morganella morganii* | 5 |
| Else | 48 |
| Multiple drug resistance | 101 |
| Carbapenem resistant *Acinetobacter baumannii* | 32 |
| Methicillin-resistant *Staphylococcus aureus* | 18 |
| Extended Spectrum *β-lactamases* | 17 |
| Carbapenem resistant *Enterobacteriaceae* | 16 |
| Methicillin-resistant Coagulase negative *Staphylococci* | 10 |
| Methicillin-resistant *Staphylococcus epidermis* | 5 |
| Carbapenem resistant *Pseudomonas aeruginosa* | 3 |
