# Supplementary material for: Effect of multiple drug resistance on total medical costs among patients with intra-abdominal infections in China
Source: PLoS One. 2018 Mar 28;13(3):e0193977. doi: 10.1371/journal.pone.0193977 (PMC5873998; doi:10.1371/journal.pone.0193977)
Supplement: S2 Table — (DOCX) [file pone.0193977.s002.docx]

| S2 Table. Isolation of sample types |  |
| --- | --- |
| Sample types | No. of isolates |
| Non-resistance | 35 |
| Drainage fluid | 10 |
| Ascites | 7 |
| Urine | 5 |
| Sputum | 3 |
| Else | 10 |
| Non-multiple drug resistance | 126 |
| Drainage fluid | 36 |
| Sputum | 21 |
| Ascites | 19 |
| Pus | 16 |
| Feces | 8 |
| Secreta | 8 |
| Urine | 6 |
| Blood | 5 |
| Else | 7 |
| Multiple drug resistance | 101 |
| Sputum | 30 |
| Pus | 17 |
| Drainage fluid | 17 |
| Feces | 10 |
| Blood | 10 |
| Ascites | 7 |
| Else | 10 |
